# Premium Teeth

**Published:** 1850-10

**Authors:** 


*Premium Teeth.*—At the late Mechanics' Fair of the Maryland Institute, a silver medal was awarded to Jones, White & Co. for the best specimen of single, plain and gum porcelain teeth, exhibited on the occasion. We are indebted to Mr. White, one of the firm, for a sample of the teeth exhibited at the Fair. They are certainly very beautiful, approaching the natural teeth as closely in appearance as any we have seen.

A diploma was also awarded to Dr. Burton of Baltimore, at the same time, for the best specimen of block teeth.

